# Imaging-Guided Therapy Simultaneously Targeting HER2 and EpCAM with Trastuzumab and EpCAM-Directed Toxin Provides Additive Effect in Ovarian Cancer Model

**DOI:** 10.3390/cancers13163939

**Published:** 2021-08-04

**Authors:** Tianqi Xu, Anzhelika Vorobyeva, Alexey Schulga, Elena Konovalova, Olga Vorontsova, Haozhong Ding, Torbjörn Gräslund, Liubov A. Tashireva, Anna Orlova, Vladimir Tolmachev, Sergey M. Deyev

**Affiliations:** 1 Department of Immunology, Genetics and Pathology, Uppsala University, 751 85 Uppsala, Sweden; tianqi.xu@igp.uu.se (T.X.); olga.vorontsova@igp.uu.se (O.V.); 2 Research Centrum for Oncotheranostics, Research School of Chemistry and Applied Biomedical Sciences, National Research Tomsk Polytechnic University, 634 050 Tomsk, Russia; schulga@gmail.com (A.S.); anna.orlova@ilk.uu.se (A.O.); biomem@mail.ru (S.M.D.); 3 Molecular Immunology Laboratory, Shemyakin & Ovchinnikov Institute of Bioorganic Chemistry, Russian Academy of Sciences, 117997 Moscow, Russia; Elena.ko.mail@gmail.com; 4 Department of Protein Science, KTH Royal Institute of Technology, Roslagstullsbacken 21, 114 17 Stockholm, Sweden; haozhong@kth.se (H.D.); torbjorn@kth.se (T.G.); 5 Cancer Research Institute, Tomsk National Research Medical Center Russian Academy of Sciences, 634009 Tomsk, Russia; tashireva@oncology.tomsk.ru; 6 Department of Medicinal Chemistry, Uppsala University, 751 23 Uppsala, Sweden; 7 Science for Life Laboratory, Uppsala University, 751 23 Uppsala, Sweden; 8 Bio-Nanophotonic Lab, Institute of Engineering Physics for Biomedicine (PhysBio), National Research Nuclear University ‘MEPhI’, 115409 Moscow, Russia; 9 Center of Biomedical Engineering, Sechenov University, 119991 Moscow, Russia

**Keywords:** targeted therapy, DARPin, EpCAM, trastuzumab, HER2, pseudomonas exotoxin A, combination

## Abstract

**Simple Summary:**

Targeted therapeutics provide cytostatic or cytotoxic action selectively to tumor cells while reducing the toxicity to normal cells. Targeting two molecular receptors overexpressed on tumor cells is a way to overcome heterogeneity of expression and improve therapeutic efficacy. Combining drugs with different modes of action might also increase the cytotoxic effect and decrease the chance for the cancer cells to develop resistance to treatment. In this work, we investigated a combination of the clinically used monoclonal antibody trastuzumab with a potent targeting protein–toxin fusion, directed at two different targets present in a large fraction of ovarian cancers. Co-targeted treatment provided a significant reduction in tumor growth and extended the survival of mice compared with the control and monotherapy groups. Our findings support further development of targeted combination therapies for treatment of aggressive and resistant cancer types.

**Abstract:**

Efficient treatment of disseminated ovarian cancer (OC) is challenging due to its heterogeneity and chemoresistance. Overexpression of human epidermal growth factor receptor 2 (HER2) and epithelial cell adhesion molecule (EpCAM) in approx. 30% and 70% of ovarian cancers, respectively, allows for co-targeted treatment. The clinical efficacy of the monoclonal antibody trastuzumab in patients with HER2-positive breast, gastric and gastroesophageal cancers makes it readily available as the HER2-targeting component. As the EpCAM-targeting component, we investigated the designed ankyrin repeat protein (DARPin) Ec1 fused to a truncated variant of Pseudomonas exotoxin A with reduced immunogenicity and low general toxicity (LoPE). Ec1-LoPE was radiolabeled, evaluated in ovarian cancer cells in vitro and its biodistribution and tumor-targeting properties were studied in vivo. The therapeutic efficacy of Ec1-LoPE alone and in combination with trastuzumab was studied in mice bearing EpCAM- and HER2-expressing SKOV3 xenografts. SPECT/CT imaging enabled visualization of EpCAM and HER2 expression in the tumors. Co-treatment using Ec1-LoPE and trastuzumab was more effective at reducing tumor growth and prolonged the median survival of mice compared with mice in the control and monotherapy groups. Repeated administration of Ec1-LoPE was well tolerated without signs of hepatic or kidney toxicity. Co-treatment with trastuzumab and Ec1-LoPE might be a potential therapeutic strategy for HER2- and EpCAM-positive OC.

## 1. Introduction

Ovarian cancer (OC) is the leading cause of death among women with cancers of the reproductive system. The majority of patients are diagnosed late due to the absence of specific symptoms before the cancer has spread outside of the pelvis and the lack of effective screening methods [1,2]. The standard treatment of OC is cytoreductive surgery in combination with platinum-based chemotherapy. An emerging trend is also to apply neoadjuvant chemotherapy before primary tumor reductive surgery [3]. However, this conventional approach is not efficient for disseminated OC and drug resistance often develops after several cycles of treatment, resulting in relapse, fast progression, and low overall survival [2,3]. The heterogeneity of OC and its chemoresistance have promoted the development of molecular targeted therapies [2,4]. An anti-angiogenesis agent, the monoclonal antibody (mAb) bevacizumab targeting vascular endothelial growth factor (VEGF), and poly(adenosine diphosphate-ribose) polymerase (PARP) inhibitors, such as olaparib, have recently been included in OC treatment guidelines [1]. Selective targeted delivery of cytotoxic payloads, such as drugs or toxins, to tumor cells might improve the safety and efficacy of OC treatment.

Potential molecular targets for OC treatment are the human epidermal growth factor receptor 2 (HER2) and the epithelial cell adhesion molecule (EpCAM), overexpressed in approx. 30% and 70% of OCs, respectively [5,6]. HER2 is an established therapeutic target in breast cancer and is under clinical investigation in OC [7]. It is a transmembrane protein with tyrosine kinase activity. HER2 gene amplification, mutation, and/or HER2 overexpression are frequently found in solid tumors and are associated with increased recurrence of disease and poor prognosis of survival in patients [8]. HER2-targeted therapy using trastuzumab prolongs survival of patients with HER2-overexpressing breast, gastroesophageal and gastric cancers [9,10,11], and has been proven effective in OC [12]. However, a major issue with trastuzumab treatment is the development of primary or acquired resistance [13]. To overcome it, combination therapies have been investigated, such as the addition of pertuzumab, a mAb also targeting HER2, or the tyrosine kinase inhibitor lapatinib, or targeted delivery of a cytotoxic drug by the antibody–drug conjugate (ADC) trastuzumab emtansine (T-DM1) [14]. Mechanisms involved in resistance to treatment are shared between HER2-targeting agents [14] and cases with a reduced number of HER2 receptors on the cancer cell membrane due to exposure to trastuzumab have been reported [15]. The heterogeneity of HER2 expression within tumors in the same patient and between patients also creates issues for HER2-targeting therapy [16].

The epithelial cell adhesion molecule (EpCAM) is a transmembrane glycoprotein that has a basal-level expression on non-pathologic epithelial cells and intense overexpression in many epithelial tumors, on cancer stem cells, and on circulating tumor cells [6,17,18]. The expression level of EpCAM on the cell surface correlates with cell adhesion, proliferation, migration, and invasion [19,20]. In epithelial OC, EpCAM expression is significantly higher in primary, metastatic, and recurrent tumors in comparison with normal ovarian tissues [21,22], and it is an independent prognostic marker for reduced survival [23]. EpCAM is an actively investigated therapeutic target in lung, gastric, colorectal, breast, and ovarian cancers [24,25]. An increase in EpCAM expression was found in tumor tissues of OC patients after platinum-based chemotherapy [26], which suggests the potential use of EpCAM-targeted therapy as a second-line treatment. EpCAM-positive stem cells in OC have a high tumorigenic potential [27], which further supports the development of anti-EpCAM therapy for preventing cancer progression [26,28].

The EpCAM-targeting therapeutic agents that have reached clinical trials are based on mAbs [29,30] and their derivatives [31]. The trifunctional mAb catumaxomab (anti-EpCAM x anti-CD3) is approved in the European Union for treatment of malignant ascites in patients with EpCAM-positive carcinomas [32]. Catumaxomab delayed ascites accumulation in a phase II/phase III clinical trial and was more effective than standard treatment (paracentesis); however, it did not impact disease progression or overall survival [30]. In another study, a combination of catumaxomab and systemic chemotherapy in patients with gastric cancer-associated peritoneal carcinomatosis was feasible and tolerable, but the primary endpoint (microscopic complete remission) was not reached [33]. The limited efficacy of the above-described approaches could have been due to several reasons: heterogeneity of target expression in patients, limited penetration of bulky mAbs into tumors, limited mode-of-action, insufficient dosing due to toxicity, and development of resistance due to inefficient treatment.

To achieve a high efficacy of targeted therapy, it is important to select a responsive subset of patients with high expression of a molecular target in the tumors. The heterogeneity of EpCAM overexpression in tumors has been documented for many cancer types [6]. Clinical trials evaluating the EpCAM-targeting mAb adecatumumab for breast cancer treatment showed that the probability of tumor progression was significantly lower in patients with tumors expressing high levels of EpCAM and receiving a high dose [29,34]. Selection of patients for targeted therapies using a biopsy-based approach could be complemented by positron emission tomography (PET) or single photon emission tomography (SPECT) imaging forming a theranostic approach for patient treatment. Imaging allows for non-invasive whole-body assessment of target expression that can be performed repeatedly over time, for example, before, during, and after treatment to monitor changes in expression or receptor occupancy.

To increase the potency of targeted therapy, a cytotoxic payload, e.g., bacterial or plant toxin, can be conjugated to a mAb or an antibody fragment creating immunotoxins. Several EpCAM-specific immunotoxins have reached clinical trials [35], such as the mouse mAb MOC31 conjugated to Pseudomonas aeruginosa exotoxin A (PE) (MOC31PE) [36,37], a Fab fragment conjugated with de-bouganin (VB6-845, citatuzumab bogatox) [38] and an scFv fragment (4D5MOCB) conjugated to Pseudomonas exotoxin A (VB4-845, oportuzumab monatox) [39,40]. The use of an engineered scaffold protein (ESP), designed ankyrin repeat protein (DARPin), for targeted delivery offers several advantages in comparison with the traditional mAbs [41]. The small size of DARPins (14–18 kDa) could provide faster extravasation and deeper penetration into the tumors compared to mAbs (150 kDa) resulting in more efficient delivery of the cytotoxic payload. Genetic engineering allows for production of DARPin-toxins as single protein fusions in high yields with lower manufacturing costs [41,42,43]. Clinical trials evaluating DARPins as VEGF-A-targeted therapeutics [44] and as HER2 imaging probes [45] did not find any toxic or immunogenic side effects associated with the protein scaffold. Additionally, DARPins have shown promising results in preclinical studies for imaging of HER2 [46,47,48,49] and EpCAM expression [50,51,52] and could be used as companion diagnostic agents during therapy.

In this study, we investigated the use of the DARPin Ec1, which binds to EpCAM with picomolar affinity (KD 68 pM [53]), as a targeting moiety for delivery of the LoPE toxin for therapy of OC. The Ec1-LoPE fusion protein (43 kDa) consists of the N-terminal DARPin Ec1 (18 kDa) and the deimmunized C-terminal catalytic subunit of PE (LoPE, 25 kDa). The binding of Ec1 to EpCAM triggers receptor-mediated endocytosis, which allows for delivery of cytotoxic agents to EpCAM-expressing cells [53,54]. Several variants of the PE toxin have been previously studied for targeted therapy including EpCAM-directed immunotherapy [55,56,57]. The exotoxin A contains several functional domains including receptor binding domain I (Ia, Ib), intracellular processing domain II, and the catalytically active domain III for toxic action. Domain III is an NAD+-diphthamide ADP-ribosyltransferase that can inactivate the eukaryotic translation elongation factor 2 (eEF2) to inhibit protein synthesis in the cell [58]. LoPE is a variant of PE, which lacks the natural receptor-binding domains Ia and Ib, most of domain II and human and murine B-lymphocyte reactive epitopes [59,60]. A LoPE fusion with DARPin 9_29 showed lower immunogenicity and general toxicity in vivo compared to a fusion between DARPin 9_29 and PE40, while efficiently suppressing the growth of HER2-expressing human OC xenografts [43]. In a recent pre-clinical study, the Ec1-LoPE fusion was effective at reducing BT-474 breast cancer xenografts [54]. We have recently shown that Ec1 specifically targets EpCAM-expressing OC xenografts and that large doses of Ec1 can be administered before saturating the uptake in SKOV3 xenografts [51].

Targeting several molecular targets overexpressed on tumor cells is a way to overcome the heterogeneity of expression and improve therapeutic efficacy. Combination therapy using drugs with different modes of action might increase the cytotoxic effect to tumor cells and decrease their chances to develop resistance while reducing the toxicity to normal tissues [61]. The goal of this study was to test a hypothesis that EpCAM-targeted delivery of LoPE toxin can improve survival of mice bearing HER2- and EpCAM-expressing SKOV3 xenografts treated with the anti-HER2 mAb trastuzumab. We first evaluated the feasibility of co-targeting HER2 and EpCAM in SKOV3 cells, studied binding, internalization, and cytotoxicity of Ec1-LoPE in vitro, and biodistribution and specificity of tumor targeting in vivo. The therapeutic efficacy of combination treatment using Ec1-LoPE and trastuzumab on tumor growth and survival of mice bearing SKOV3 xenografts was compared with the efficacy of monotherapies.

## 2. Results

### 2.1. Protein Production and Characterization

**Figure 1:**
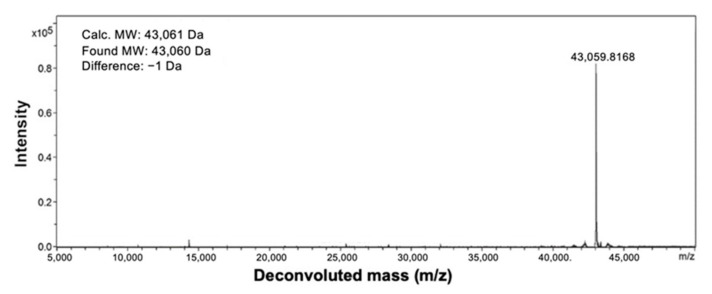
ESI-MS analysis of Ec1-LoPE fusion protein. The observed molecular weight is 43,060 Da (calculated value 43,061 Da).

The Ec1-LoPE fusion protein was produced in E. coli as described by Shramova et al. [54]. Analysis by mass spectroscopy showed a protein with a molecular weight of 43,060 Da, which was within 1 Da of the expected molecular weight (43,061 Da) (Figure 1).

### 2.2. Radiolabeling

To provide a residualizing label for evaluation of cellular processing and internalization, Ec1-LoPE was labeled with [^99m^Tc][Tc(CO)_3_(H_2_O)_3_]^+^. The labeling resulted in a radiochemical yield of 52 ± 14% (*n* = 6). The radiochemical purity after size-exclusion purification was over 99% (*n* = 6). The use of the residualizing ^99m^Tc label provides good intracellular retention of activity and, therefore, is the best choice for internalization studies.

To study the specificity and affinity of binding to EpCAM-expressing cells, Ec1-LoPE and trastuzumab were labeled with iodine-125. Indirect radioiodination using *para*-iodobenzoate ([^125^I]I-PIB) provided [^125^I]I-PIB-Ec1-LoPE in 15 ± 4% (*n* = 2) radiochemical yield. After purification, the purity was over 97% for both compounds. Direct radioiodination provided over 95% radiochemical yield for both [^125^I]I-Ec1-LoPE and [^125^I]I-trastuzumab. To image HER2 expression in vivo, the ZHER2:V2 affibody molecule was labeled with technetium-99m with a radiochemical yield of 98% (*n* = 1).

### 2.3. In Vitro Specificity

**Figure 2:**
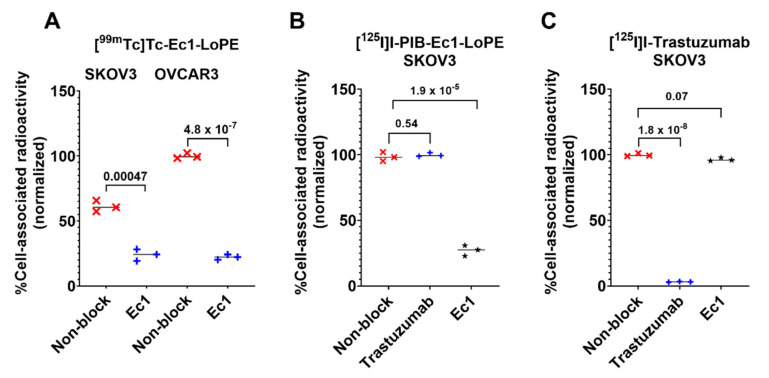
In vitro binding specificity of (**A**) [^99m^Tc]Tc-labeled Ec1-LoPE in SKOV3 and OVCAR3 cells expressing EpCAM; Symbols (×) show individual measured values for binding to non-blocked cells, and symbols (+) measured values for binding to blocked cells. (**B**) cross-blocking study of [^125^I]I-labeled Ec1-LoPE; and (**C**) [^125^I]I-labeled trastuzumab in EpCAM- and HER2-expressing SKOV3 cells. For blocking, a 100-fold molar excess of anti-EpCAM DARPin Ec1 or anti-HER2 antibody trastuzumab was added to blocked groups. The final concentration of radiolabeled compound was 2 nM. The data are presented as average ± SD (*n* = 3). *p*-values from unpaired *t*-test (equal variation) are provided to characterize differences between activity uptake in blocked and non-blocked groups. Symbols (×) show individual measured values for binding to non-blocked cells, symbols (+) measured values for binding to cells blocked with trastuzumab, and symbols (★) measured values for binding to cells blocked with Ec1.

The binding specificity of [^99m^Tc]Tc-labeled Ec1-LoPE was tested using EpCAM-expressing SKOV3 and OVCAR3 ovarian cancer cell lines. By pre-saturation of EpCAM with the non-labeled Ec1, the uptake of radioactivity was significantly decreased (*p* < 0.05, *t*-test) (Figure 2A), which indicated EpCAM-mediated binding of [^99m^Tc]Tc -labeled Ec1-LoPE to the cells.

To test if Ec1 interferes with the binding of trastuzumab to HER2 or if trastuzumab interferes with the binding of Ec1 to EpCAM, a cross-blocking experiment was performed using SKOV3 cells, which express both receptors (Figure 2B,C). Compared to the non-blocked group, the uptake of [^125^I]I-Ec1-LoPE was significantly (*p* < 0.05, *t*-test) lower when EpCAM was blocked with Ec1 but not when HER2 was blocked with trastuzumab (Figure 2B). A significant (*p* < 0.05, *t*-test) decrease in uptake of [^125^I]I-trastuzumab was observed when HER2 was blocked with trastuzumab but not when EpCAM was blocked with Ec1 (Figure 2C). These results demonstrated that Ec1-LoPE and trastuzumab recognize EpCAM and HER2, respectively, and do not have a cross-blocking effect.

### 2.4. Cellular Processing of Ec1-LoPE

**Figure 3:**
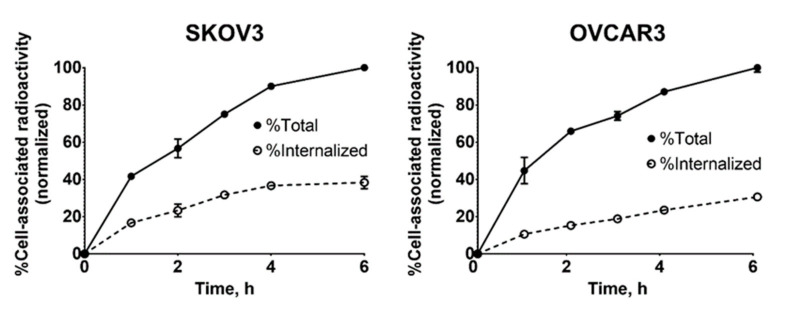
Cellular processing and internalization of [^99m^Tc]Tc-Ec1-LoPE in EpCAM-expressing cell lines SKOV3 and OVCAR3. The data are presented as the average of three samples ± SD.

To understand the rate of cellular processing and internalization, SKOV3 and OVCAR3 cells were incubated with [^99m^Tc]Tc-labeled Ec1-LoPE for 6 h and analyzed for total cell-associated and internalized activity using the acid wash test (Figure 3). A similar pattern was observed for the SKOV3 and OVCAR3 cell lines. The internalization was rapid with relatively small variation between cell lines. Continuous incubation resulted in a slow increase in cell-bound activity of [^99m^Tc]Tc-labeled Ec1-LoPE over time and the internalized fraction at 6 h reached 38 ± 3% of total cell-associated activity for SKOV3 and 31 ± 0% for OVCAR3 cells.

### 2.5. Affinity Measurement

**Figure 4:**
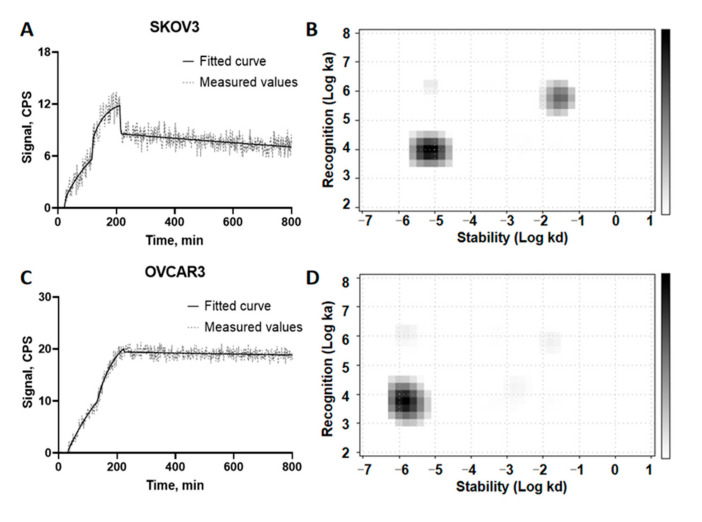
Binding kinetics of [^125^I]I-Ec1-LoPE to (**A**) SKOV3 cells and (**C**) OVCAR3 cells analyzed by TraceDrawer and corresponding interaction maps (**B**,**D**). Data are representatives from duplicates.

Evaluation of the binding kinetics of [^125^I]I-Ec1-LoPE binding to SKOV3 and OVCAR3 cells was performed in real-time using a LigandTracer Grey instrument. Rapid binding and slow dissociation were observed for both cell lines on the TraceDrawer sensorgrams (Figure 4A,C). The Interaction Map analysis (Figure 4B,D) indicated two interactions, a major high-affinity one and a minor of lower affinity, for both cell lines. The corresponding equilibrium dissociation constants (KD) and weight of each interaction (%) are shown in Table 1.

### 2.6. In Vitro Cytotoxicity

**Figure 5:**
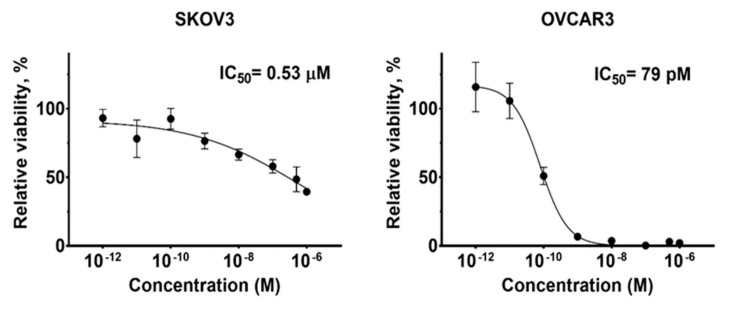
Cytotoxicity of Ec1-LoPE in EpCAM-expressing SKOV3 and OVCAR3 cell lines. The cells were incubated with dilution series of the Ec1-LoPE DARPin-toxin (0–1000 nM). The viability of cells grown in media without the toxin was used as 100% viability control. The viability curve is plotted with relative viability as *y*-axis and concentration of Ec1-LoPE as *x*-axis. The data are presented as average ± SD (*n* = 4−6).

The cytotoxicity of Ec1-LoPE was measured by treating EpCAM-expressing SKOV3 and OVCAR3 cells with serial dilutions of Ec1-LoPE followed by measurement of cell viability (Figure 5). Ec1-LoPE demonstrated a dose-dependent cytotoxic effect with a subnanomolar IC50 value (79 pM) on OVCAR3 cells and a submicromolar IC50 value (0.53 µM) on SKOV3 cells.

### 2.7. Biodistribution Study

**Figure 6:**
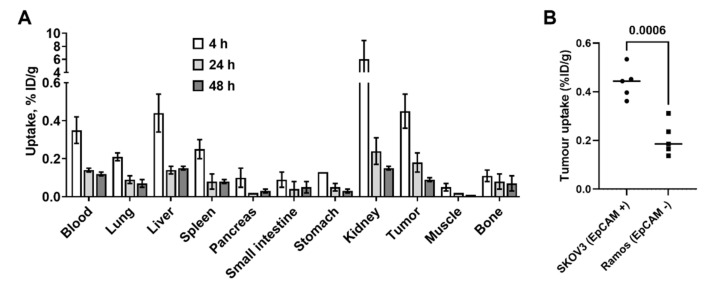
Biodistribution of [^125^I]I-PIB-Ec1-LoPE in female BALB/c nu/nu mice. (**A**) Biodistribution of ^[125^I]I-PIB-Ec1-LoPE in mice bearing SKOV3 xenografts at 4, 24, and 48 h post-injection (pi). The uptake values are presented as an average from three to five animals ± SD. (**B**) Comparison (*t*-test) of ^[125^I]I-PIB-Ec1-LoPE uptake in EpCAM-positive SKOV3 xenografts and EpCAM-negative Ramos xenografts 4 h pi. Difference between uptake in other organs in mice bearing SKOV3 (•) and Ramos (▪) xenografts was not significant (*p* > 0.05, *t*-test).

To determine the biodistribution of Ec1-LoPE over time, female BALB/c nu/nu mice bearing SKOV3 xenografts were injected with [^125^I]I-PIB-Ec1-LoPE. The animals were euthanized at 4, 24, and 48 h pi, the organs and tissues were excised, and their radioactivity was measured (Figure 6A). Biodistribution was characterized by a low level of activity retention in the majority of normal organs (below 1%ID/g at 4 h) except kidneys. Fast clearance from blood was observed, as the radioactivity in blood was less than 0.5% ID/g after 4 h. The activity in normal organs and tumors decreased further by 24 h. Biodistribution of [^125^I]I-PIB-Ec1-LoPE in BALB/c nu/nu mice bearing EpCAM-positive SKOV3 xenografts and EpCAM-negative Ramos xenografts was compared at 4 h pi (Figure 6B). No significant differences in uptake in normal organs or tissues (except bone, *p* = 0.018, *t*-test) were observed; however, a significantly (*p* < 0.005, *t*-test) lower uptake was observed in HER2-negative Ramos xenografts compared to HER2-positive SKOV3 xenografts.

### 2.8. Imaging of EpCAM and HER2 Expression in Mice Bearing EpCAM- and HER2-Expressing SKOV3 Tumors

**Figure 7:**
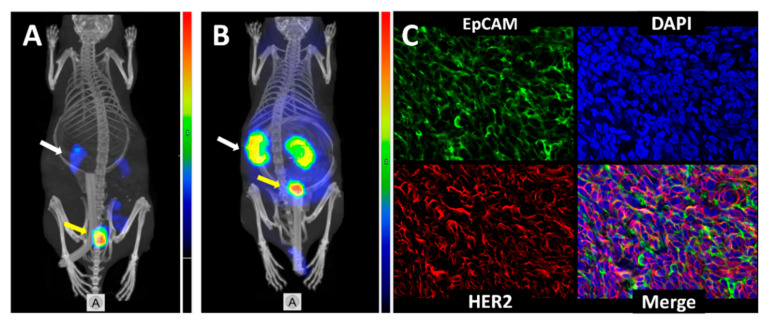
Micro-single photon emission computed tomography (SPECT)/CT imaging of (**A**) EpCAM expression using [^125^I]I-PIB-Ec1 at 6 h pi; (**B**) HER2 expression using [^99m^Tc]Tc-ZHER2:V2 at 4 h pi in BALB/c nu/nu mice bearing SKOV3 xenografts. White arrows point at kidneys, yellow arrows point at tumors. Co-expression of HER2 and EpCAM in SKOV-3 xenografts (**C**). EpCAM is stained green, HER2 is stained red, nuclei were counterstained with DAPI (blue) (200× Magnification).

SPECT/CT imaging of EpCAM expression using DARPin [^125^I]I-PIB-Ec1 (Figure 7A) and HER2 expression using affibody molecule [^99m^Tc]Tc-ZHER2:V2 (Figure 7B) was performed in mice bearing SKOV3 xenografts. Imaging enabled clear visualization of small tumors in both cases. High accumulation of activity confirmed EpCAM and HER2 expression in tumors in the control group (from therapy study as described below). Co-expression of HER2 and EpCAM in SKOV-3 xenografts was confirmed by multiplex immunohistochemistry analysis (Figure 7C).

### 2.9. Therapeutic Effect in Mice Bearing EpCAM- and HER2-Expressing SKOV3 Tumors

**Figure 8:**
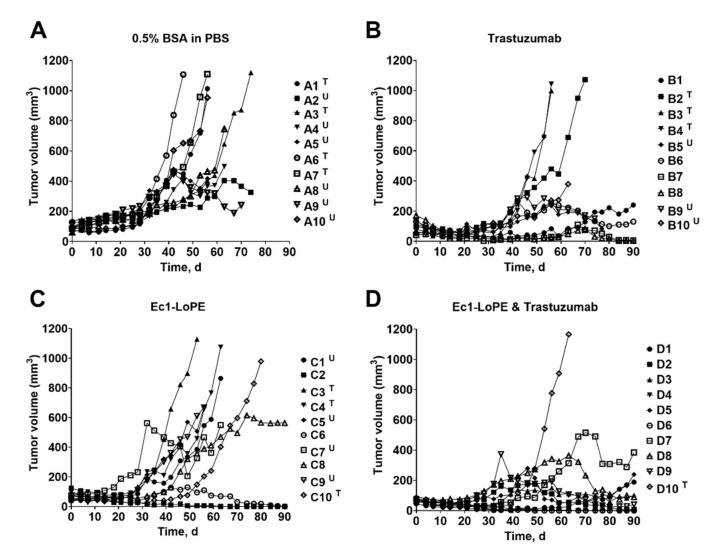
Tumor volume growth curves for individual mice from four groups receiving: subcutaneous (s.c.) injection of vehicle (0.5% BSA in PBS) for six consecutive weeks (**A**), s.c. injection of trastuzumab (**B**), intravenous (i.v.) injection of Ec1-LoPE (**C**), and s.c. injection of trastuzumab together with i.v. injection of Ec1-LoPE (**D**). The doses and frequency of trastuzumab injections were 8 mg/kg for the first two weeks and 4 mg/kg for the next four consecutive weeks. The injections of Ec1-LoPE (1 mg/kg) were carried out at days 0, 2, 4, 7, 35, 37, 39, 42. Mice were euthanized when xenograft volume exceeded 1000 mm^3^ (T) or bleeding ulcers were observed (U). Four groups of mice (10 animals per group) were used in the experiment.

**Figure 9:**
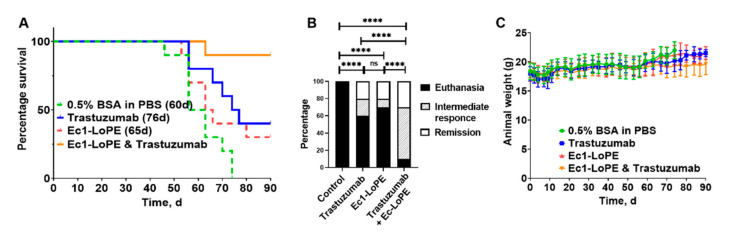
Antitumor effect of monotherapies using Ec1-LoPE or trastuzumab and their combination in female BALB/c nu/nu mice bearing EpCAM- and HER2-expressing SKOV3 xenografts. Four groups of mice (10 animals per group) were used in the experiment. (**A**) Survival of mice during the experiment. (**B**) Therapy outcomes for different treatment groups. The outputs categories were defined as euthanasia (animal reached humane endpoint before the study termination), intermediate response (macroscopic tumors at the study termination), and remission (no tumors at the study termination). The difference between groups was determined using chi-square test. Symbol **** corresponds to *p* < 0.0001. (**C**) Average animal weight.

The anti-tumor effect of EpCAM-targeting treatment with Ec1-LoPE alone or in combination with the HER2-targeting mAb trastuzumab was evaluated in BALB/c nu/nu mice bearing EpCAM- and HER2-expressing SKOV3 xenografts. To evaluate the effect of a combination treatment, one group receiving monotherapy with trastuzumab was included. Mice receiving vehicle solution (0.5% BSA in PBS) were used as a control. The tumor volume was monitored twice every week and the tumor growth curve for each mouse is shown in Figure 8A–D. The tumors in the control group grew rapidly. Four out of ten mice (40%) were euthanized when tumors reached the size limit and six out of ten (60%) were euthanized due to tumor ulceration. The median survival in this group was 60 days (Figure 9A).

In the single treatment groups, where mice were treated with Ec1-LoPE alone or trastuzumab alone, the tumor growth was inhibited but regrowth occurred after the treatment was discontinued. Six out of ten mice (60%) in the trastuzumab treatment group and seven out of ten (70%) in the Ec1-LoPE treatment groups were euthanized because of tumor size limit or surface ulceration (Figure 9B). Both groups had two out of ten mice (20%) with complete regression with no macroscopically detectable tumors at the study termination (90 days), which was the maximum study length according to the ethical permit. In the co-treatment group, the tumor size was significantly (*p* < 0.05, one-way ANOVA, Bonferroni analysis) smaller compared to the control group, starting already from day four (Figure S1) until day ninety (Figure S2). By the end of the experiment, three out of ten mice (30%) had complete tumor regression and two out of ten (20%) had a tumor size below 100 mm^3^.

The median survival time in the treatment groups with single therapy was longer than in the vehicle control group (60 days for control, 65 days for Ec1-LoPE, and 76 days for trastuzumab). In the combination treatment group, only one mouse was euthanized within the permitted follow-up time of 90 days, resulting in significantly prolonged survival in the co-treatment group compared to the single treatment groups (Mantel–Cox log-rank test, *p* = 0.0083 compared to Ec1-LoPE monotherapy and *p* = 0.026 compared to trastuzumab monotherapy) and the control group (Mantel–Cox log-rank test, *p* = 0.0001). Therapy outcome was significantly better for the combination therapy than for monotherapies according to chi-square test (Figure 9B).

The weight of the animals was monitored and the differences between the groups were within standard deviation (Figure 9C). No tendency for weight loss was observed in any of the treated groups when compared to the control group, indicating that the treatments were well tolerated. Post-mortem histologic examination of livers and kidneys of mice that received mono- or co-treatment with Ec1-LoPE and/or trastuzumab did not find significant morphologic differences or signs of injury (Figure S3).

## 3. Discussion

Protein-based targeted therapeutics in comparison with conventional chemotherapeutic drugs contain a targeting moiety that can deliver a conjugated/fused toxin or a drug specifically to the tumor cells while reducing the off-target toxicity to normal cells [62]. Dual targeting or co-targeting strategies offer the possibility to focus the cytotoxic effect on cancer cells through a combination of agents with different modes of action while spreading out toxic side-effects due to different toxicity profiles. They could also potentially help to overcome some mechanisms of resistance that may develop to a monotherapy [61]. One of the co-targeting strategies currently used in clinical practice includes targeting of different epitopes on the same receptor overexpressed on cancer cells, e.g., the two mAbs trastuzumab and pertuzumab, both targeting HER2, as first-line treatment of advanced breast cancer [63]. A growing number of approaches are being evaluated in clinical trials, e.g., the use of bispecific antibodies targeting two different molecular targets [64].

Overexpression of HER2 and EpCAM in a large number of carcinomas, including OC, make them attractive targets for dual targeting. Co-expression of HER2 and EpCAM correlates with a poor prognosis, e.g., in breast cancer [65], which additionally supports the development of co-targeted therapies for these patients. Analysis of mRNA suggests very high level of EpCAM and elevated level of HER2 expression in high-grade serous ovarian cancer [66]. However, the data concerning *ERBB2* gene amplification or level of HER2 protein expression are not provided in that study. Our extensive literature search did not reveal clinical data concerning co-expression of HER2 and EpCAM proteins in ovarian cancer, and we need to make estimations. The most reliable predictive biomarker for response to anti-HER2 therapy is an amplification of the *ERBB2* gene. Results of amplification measurements are much less dependent on interpretation than the results of immunohistochemistry analysis of protein expression. The highest frequency of the *ERBB2* gene overexpression was found in mucinous ovarian carcinoma, between 19% and 26.7% [67,68]. This gene is also overexpressed in 14% of ovarian clear cell carcinomas [69]. Taking into account that high overexpression of EpCAM is observed in 85% of mucinous ovarian carcinomas and in 90% of ovarian clear cell carcinomas [70], there must be tumors of these histotypes with high levels of both HER2 and EpCAM expression. 

The clinical efficacy of trastuzumab in patients with HER2-positive breast, gastric and gastroesophageal cancers makes it a readily available option as a HER2-targeting therapeutic component. EpCAM, a well-known biomarker of circulating tumor cells and cancer stem cells [18], has been actively investigated as a therapeutic target using mAbs in primary or adjuvant settings; however, this has displayed limited clinical efficacy [35]. Factors limiting clinical efficacy of EpCAM-targeting antibodies include poor penetration in tumor mass, insufficient anti-tumor action, and lack of patient stratification to identify potential responders prior to therapy [35]. In this study, we used EpCAM-targeting DARPin Ec1. The molecular mass of this small protein is approximately eight-fold smaller than the mass of an IgG antibody, i.e., it is appreciably smaller. Even if fused with a toxin, Ec1 is smaller than a Fab fragment of an antibody.

To enhance the cytotoxic effect of EpCAM-targeted therapy, various toxins have been conjugated to mAbs or their fragments. In a study by Stish et al., a bispecific immunotoxin targeting HER2 and EpCAM had higher activity than the monospecific immunotoxin targeting HER2 in several xenograft models in mice [70]. However, significant side effects, such as weight loss, renal and hepatic damage were observed. Patients treated with the first-generation exotoxin A (ETA)-based immunotoxins developed vascular leak syndrome (VLS) due to killing of vascular endothelial cells by the toxin [71]. The second-generation ETA-immunotoxins with a deleted cell-binding domain had weaker binding to normal tissues, but the VLS symptoms remained [72,73,74,75]. To avoid systemic toxicity, some clinical studies investigated local administration of a scFv-PE fusion (VB4-845) to patients with squamous cell carcinoma of the head and neck (intratumoral) and transitional cell carcinoma of the bladder (intravesical) [76]. Reduction of the size of the targeted agent might have two beneficial effects on therapy. First, smaller agents have a shorter residence in circulation, which reduces the exposure of vascular endothelial cells and has the potential to decrease systemic toxicity. Second, smaller proteins penetrate tumors more rapidly. Several EpCAM-targeting DARPins conjugated to PE40 (ETA”) were developed and showed strong anti-tumor responses in vivo: Ec4-ETA” [77] in HT-29 colon cancer cells and in SW2 small cell lung carcinoma xenografts, Ec1-ETA” and its PEGylated version PEG20kDa-Ec1-ETA” [78] in MDA-MB-468 breast cancer xenografts. Another PEGylated version, Ec1-ETA”486Aha-AhaKDEL-3C-PEG, was developed and had lower systemic toxicity in mice than the unPEGylated protein [79]. Due to their bacterial origin, protein toxins cause an immunogenic response after repeated administration in patients [80]. Identification and removal of B- and T-cell recognition epitopes on the PE38 toxin provided variants with reduced immunogenicities, such as PE38 × 8 and LoPE (PE25) [59,60,79,81,82,83]. The DARPin 9_29-LoPE fusion showed both lower toxicity and immunogenicity in vivo compared to the DARPin 9_29-PE40 fusion protein and effectively suppressed tumor growth [43]. In a recent study, the Ec1-LoPE fusion protein demonstrated significant inhibition of tumor growth in a BT-474 breast cancer model, and its combination with HER2-targeting liposomes coated with DARPin 9_29 and loaded with the ribonuclease Barnase led to the elimination of both primary tumors and metastases [54]. Ten cycles of treatment with Ec1-LoPE did not cause any observable toxicities or weight loss in mice.

In this work, we have investigated the use of Ec1-LoPE as the EpCAM-targeting component and the mAb trastuzumab as the HER2-targeting component for combination therapy of mice bearing HER2- and EpCAM-expressing SKOV3 ovarian cancer xenografts. A prerequisite for combination therapy is the absence of interference between the targeting agents for binding to the targets, which was successfully demonstrated in vitro (Figure 2B,C).

Ec1-LoPE showed EpCAM-mediated binding and a strong affinity with a subnanomolar K_D_ value to SKOV3 and OVCAR3 cells (Figure 4A, Table 1). A high internalization rate of Ec1-LoPE (Figure 3) suggested efficient intracellular delivery of LoPE and its inhibition of growth of EpCAM-overexpressing SKOV3 and OVCAR3 cells (Figure 5) demonstrated a potent cytotoxic effect in vitro. The higher IC_50_ value in SKOV3 cells compared to OVCAR3 cells might be due to several factors, such as differences in EpCAM expression level [84] and differences in sensitivity to toxin treatment. It was previously observed for HER2-targeting affibody–drug and affibody–toxin conjugates that SKOV3 cells are more resistant to cytotoxic action despite high expression of HER2 receptors [82,85,86]. The results of the in vivo specificity experiment demonstrated EpCAM-mediated accumulation of Ec1-LoPE in human xenografts in mice (Figure 6).

The lack of patient stratification was identified as a possible cause for lack of efficacy in clinical trials of EpCAM-targeting therapeutics [35]. The SPECT/CT imaging (Figure 7) was used to confirm the co-expression of EpCAM and HER2 in the used in vivo model (SKOV3 xenografts). In this study, we have demonstrated that the use of radiolabeled ESP, Ec1 DARPin and anti-HER2 affibody molecule permits visualization of target expression within a few hours after injection (Figure 7). This provides a non-invasive tool for efficient stratification of patients for the combined therapy. Accurate determination of EpCAM and HER2 before therapy would permit the exclusion of patients who have tumors with insufficient target expression and avoid overtreatment.

The experimental therapy demonstrated that Ec1-LoPE efficiently inhibited tumor growth and its combination with trastuzumab provided an additive effect (Figure 8 and Figure 9). Monotherapy with trastuzumab extended the median survival of mice from 60 days in the control group to 76 days and mono-therapy with Ec1-LoPE extended the median survival from 60 to 65 days (Figure 9A). The median survival of mice in the co-treatment group with trastuzumab and Ec1-LoPE was not reached until the end of the study (90 days), meaning a significantly prolonged survival of mice bearing SKOV3 xenografts. In each mono-treatment group, more than six mice out of ten were sacrificed and only two mice (20%) had complete tumor regression. The co-treatment with trastuzumab and Ec1-LoPE efficiently inhibited tumor growth, with two mice (20%) having a tumor size below 100 mm^3^ and three (30%) having complete regression of the tumors. Only one mouse (10%) was sacrificed due to large tumor size in the co-treatment group. Tumor re-growth was observed in all treatment groups (Figure 8B–D), but the time to relapse was delayed in the co-treatment group compared with the mono-treatment groups. Overall, the combined therapy provided significantly better therapy outcome than any monotherapy according to chi-square test (Figure 9B).

All treatments were well tolerated, without any signs of toxicity or tendency for weight loss in any of the treatment groups during the whole study period (Figure 9C). Post-mortem histologic examination did not report any evidence of hepatic toxicity (Figure S3). For livers of the mice in the treatment groups, scattered mitoses were found among hepatocytes, indicating an increased frequency of cell division as a result of regenerative activity or hyperplasia. Some livers of the mice in the treatment groups showed rounded cytoplasmic vacuoles adjacent to the cell nucleus, suggesting intracellular edema. These changes were reversible and were also observed in the control group. The kidneys were also examined for signs of toxicity or injury, but no evidence was found (Figure S3). This is in agreement with the anti-HER2 DARPin 9_29-LoPE study by Sokolova et al., where LoPE demonstrated significantly lower general toxicity compared with PE40 [43].

The co-targeting approach presented here uses targeting agents with non-overlapping toxicity profiles. The dose-limiting organs (heart for trastuzumab, liver and kidneys for Ec1-LoPE) are different due to different patterns of target expression and metabolic pathways. Therefore, the co-targeting strategy might spread the toxic side effects between healthy organs while providing an additive cytotoxic effect to tumors and overcoming the heterogeneity of target expression.

## 4. Materials and Methods

### 4.1. General Materials and Instruments

Sodium iodide [^125^I]NaI was from PerkinElmer Sverige AB (Upplands Väsby, Sweden). The kits for production of tricarbonyl technetium were purchased from the Center for Radiopharmaceutical Sciences (PSI, Villigen, Switzerland). Silica gel ITLC strips (Varian, Lake Forest, CA, USA) were used for measurements of radiochemical yield and purity and measured using Cyclone Storage Phosphor System (PerkinElmer, Waltham, MA, USA). The activity was measured using an automatic gamma spectrometer (2480 Wizard, Wallac, Finland).

The human cancer cell lines SKOV3 (primary human ovarian carcinoma) and OVCAR3 (human ovarian adenocarcinoma) expressing EpCAM receptors, as well as EpCAM-negative Ramos (Burkitts lymphoma) cell line were purchased from the American Type Culture Collection (ATCC). The cells were cultured in Roswell Park Memorial Institute (RPMI) 1640 medium supplemented with 10% fetal bovine serum (FBS), 2 mM L-glutamine, and a mixture of penicillin 100 IU/mL and 100 µg/mL streptomycin (SKOV3 cells) or 20% fetal bovine serum (FBS), 2 mM L-glutamine, 0.01 mg/mL bovine insulin and a mixture of penicillin 100 IU/mL and 100 µg/mL streptomycin (OVCAR3 cells). Cells were cultured at 37 °C and 5% CO2, unless stated otherwise. Cells were detached using trypsin-EDTA solution (0.25% trypsin, 0.02% EDTA in buffer).

### 4.2. Protein Production and Characterization

Ec1-LoPE was produced as described by Shramova et al. [54]. Briefly, the Ec1 nucleotide sequence was deduced from its amino acid sequence, published by Stefan et al. [53], taking into account the codon usage in highly expressed *E. coli* genes. The Ec1 gene was assembled from chemically synthesized overlapped oligonucleotides of 50 bp length by PCR and placed into the plasmid pDARP-LoPE between restriction sites *Nde*I and *Eco*RI. The resultant plasmid pET22-Ec1-LoPE was used to transform *Escherichia coli* BL21(DE3). The recombinant strain was grown in autoinduction medium ZYM-5052, containing 100 µg/mL kanamycin at 25 °C. The cells were harvested by centrifugation at 10,000× *g* at 4 °C for 20 min and resuspended in lysis buffer (200 mM Tris-HCl, 500 mM sucrose, 1 mM EDTA (pH 8.0), 1 mM PMSF and 60 µg/mL lysozyme). The suspension was diluted 2-fold with distilled water and incubated at room temperature for 30 min. Cells were broken on ice using a Vibra Cell ultrasonic liquid processor VCX130 (Sonics and Materials, Inc., Newtown, CT, USA). The cellular debris was pelleted at 70,000× *g* at 4 °C for 30 min. After addition of imidazole (30 mM) and NaCl (500 mM), the supernatant was filtered through a 0.22 µm membrane and applied onto a HisTrap HP, 1 mL column (Sativa, Uppsala, Sweden), equilibrated with 20 mM NaPi (pH 7.5), 500 mM NaCl and 30 mM imidazole. The bound proteins were eluted with a linear imidazole gradient 30–500 mM. The combined fractions containing Ec1-LoPE were diluted 5-fold with 25 mM Tris-Cl (pH 8.0) and loaded onto a Mono Q 10/100 GL column (Sativa), equilibrated with the same buffer. The bound proteins were eluted with a linear NaCl gradient 0–1 M. The fractions were analyzed by 15% reducing SDS-PAGE. Protein concentration was determined by UV spectroscopy using ε_280_ = 48,220 M^−1^ cm^−1^. The molecular weight of Ec1-LoPE was determined by electrospray ionization mass spectrometry using an Impact II instrument (Bruker, Billerica, MA, USA).

### 4.3. Radiolabeling

Site-specific radiolabeling of Ec1-LoPE with [^99m^Tc][Tc(CO)_3_(H_2_O)_3_]^+^ via C-terminal amino acid sequence (His)_6_ was performed as described earlier [87]. In brief, technetium-99m pertechnetate, [^99m^Tc]TcO_4_^−^, was obtained from a commercial ^99^Mo/^99m^Tc generator (Mallinckrodt, Petten, The Netherlands). The [^99m^Tc]TcO_4_^−^ eluate in 500 μL of 0.9% NaCl (3–4 GBq) was added to a CRS kit vial, followed by incubation at 100 °C for 30 min and cooling down at room temperature for 10 min to generate the [^99m^Tc][Tc(CO)_3_(H_2_O)_3_]^+^ (tricarbonyl technetium) precursor. Solution of [^99m^Tc][Tc(CO)_3_(H_2_O)_3_]^+^ (approximately 100 MBq, 15 μL) was mixed with 30 μL 0.1 M HCl and added to 30 μg (25 μL, PBS) of Ec1-LoPE, and the mixture was incubated at 40 °C for 60 min. Then, a 1000-fold molar excess of histidine (110 μg in 11 μL of PBS) was added to the radiolabeled Ec1-LoPE and incubated at 40 °C for 10 min to remove any loosely bound tricarbonyl technetium. The radiolabeled Ec1-LoPE was separated from [^99m^Tc][Tc(CO)_3_(H_2_O)_3_]^+^ by passage through a NAP-5 size exclusion column (GE Healthcare, Amersham, UK) pre-equilibrated and eluted with 1% BSA in PBS. For in vivo imaging of HER2 expression, labeling of ZHER2:V2 with technetium-99m was performed as previously described [88]. Radiochemical yield and purity of [^99m^Tc]Tc-labeled compounds were measured using radio-iTLC in PBS. In this system, the radiolabeled proteins stay at the application point, and all forms of free radionuclides move with the solvent front.

Radioiodination of Ec1-LoPE and Ec1 (for in vivo imaging of EpCAM expression) using N-succinimidyl-para-(trimethylstannyl)-benzoate was performed as described earlier [52]. Briefly, a solution of N-succinimidyl-*p*-(trimethylstannyl)-benzoate in 5% acetic acid in methanol (5 µL, 13 nmoles, 5 µg,) and 0.3% acetic acid in water (20 µL) was mixed with 10 µL of radioiodine stock solution (20 MBq). Chloramine-T (80 µg, 10 µL, 8 mg/mL in water) was added and after 5 min at room temperature sodium metabisulfite (120 µg, 10 µL, 12 mg/mL in water) was added to stop electrophilic radioiodination. After that, 70 µg of Ec1 or 150 µg of Ec1-LoPE in 150 µL of 0.07 M borate buffer (pH 9.3) were added. The conjugation reactions proceeded at room temperature for 60 min. The radiolabeled [^125^I]I-PIB-Ec1-LoPE and [^125^I]I-PIB-Ec1 were purified on a NAP-5 column, pre-equilibrated and eluted with 1% BSA in PBS.

Direct radioiodination of Ec1-LoPE and trastuzumab was performed as described in Deyev et al. [47]. To a mixture of Ec1-LoPE (50 µg, 1.2 nmol) and [^125^I]NaI (12 MBq, 4 µL) in 55 µL of PBS, chloramine T (5 µL of 2 mg/mL in PBS, 10 µg, 35 nmol) was added to start the reaction. After 60 sec incubation at room temperature, sodium metabisulfite (10 µL of 2 mg/mL in water, 20 µg, 105 nmol) was added. To a mixture of trastuzumab (25 µg, 0.17 nmol) and [^125^I]NaI (7 MBq, 3 µL), chloramine T (10 µL of 4 mg/mL in PBS, 40 µg, 138 nmol) was added to start the reaction. After 60 sec incubation at room temperature, sodium metabisulfite (10 µL of 8 mg/mL in water, 80 µg, 421 nmol) was added. The radiolabeled [^125^I]I-Ec1-LoPE was purified on a NAP-5 column pre-equilibrated and eluted with 1% BSA in PBS. The radiochemical yield and radiochemical purity after NAP-5 purification of [^125^I]I-labeled compounds were determined using radio-iTLC analysis in 80% acetone in water.

### 4.4. In Vitro Binding Specificity

Specificity of [^99m^Tc]Tc-Ec1-LoPE binding to EpCAM was tested using ovarian cancer cell lines SKOV3 and OVCAR3 following a previously described method [85]. Cells were seeded in 6-well plates at a density of 5 × 10^5^ cells per well one day before the experiment. Non-radiolabeled Ec1 (200 nM) was added to one group of three cell dishes to saturate the EpCAM receptors and an equal volume of medium only was added to the second group of three dishes followed by incubation for 15 min at room temperature. Then, a solution of ^[99m^Tc]Tc-Ec1-LoPE was added to each dish to reach a final concentration of 2 nM followed by incubation for 1 h at 37 °C. After incubation, the medium was collected and the cells were detached and collected, followed by washing with PBS. The activity in fractions containing medium and cells was measured using a gamma-spectrometer and cell-associated activity was calculated.

To evaluate the feasibility of co-targeting HER2 and EpCAM receptors in vitro, a cross-blocking of [^125^I]I-Ec1-LoPE binding by trastuzumab and [^125^I]I-trastuzumab binding by Ec1 were evaluated. Non-radiolabeled Ec1 (200 nM) was added to one group of 3 cell dishes and trastuzumab (200 nM) was added to another group of 3 cell dishes to saturate the receptors. An equal volume of medium only was added to the third group of the dishes followed by incubation for 30 min at room temperature. Then, a solution of a radiolabeled Ec1-LoPE or trastuzumab was added to all groups to reach a final concentration of 2 nM, followed by incubation for 1 h at 37 °C. The cell-associated activity was measured as described above.

### 4.5. Cellular Processing of Ec1-LoPE

Cellular processing of Ec1-LoPE after binding to SKOV3 and OVCAR3 cells was studied during continuous incubation with technetium-99m-labeled Ec1-LoPE as described by Wållberg and Orlova [89]. The residualizing [^99m^Tc]Tc-label was selected for this experiment because its radiometabolites do not diffuse from cells after lysosomal degradation of internalized proteins. Thus, it represents the internalized activity in the best way. Cells were seeded at a density of 7 × 10^5^ cells per dish in 3 cm Petri dishes. The next day, [^99m^Tc]Tc-Ec1-LoPE (1 nM) was added to the cells and the cells were incubated at 37 °C, 5% CO2 in a humidified incubator. At 1 h, 2 h, 4 h, and 6 h the membrane-bound fraction was collected in a set of three dishes after treatment with 0.2 M glycine buffer containing 4 M urea (pH 2.0). The internalized fraction was determined by collecting the cell lysates after treatment with 1 M NaOH for 30 min, and activity in each fraction was measured.

### 4.6. Affinity Measurement

The binding affinity of [^125^I]I-Ec1-LoPE to SKOV3 and OVCAR3 cells was measured using LigandTracer Grey Instrument (Ridgeview Instruments, Vänge, Sweden) as described previously [51].

Briefly, one day before the experiment, cells were seeded in one sector of a Petri dish. The experiment was performed at room temperature. Two separate measurements were performed for each cell line. The kinetics of binding of [^125^I]I-Ec1-LoPE to the cells was measured continuously after adding the labelled compound to obtain concentrations of 11 and 33 nM. Thereafter, the cell culture medium was replaced by a medium not containing [^125^I]I-Ec1-LoPE and dissociation rate was measured. The equilibrium dissociation constants (K_D_) were calculated using the TraceDrawer Software (Ridgeview Instruments, Vänge, Sweden). Based on association and dissociation rate data, the equilibrium dissociation constants (K_D_) were calculated using the TraceDrawer Software (Ridgeview Instruments, Vänge, Sweden). Interaction heterogeneity was estimated using interaction map analysis (Ridgeview Diagnostics, Uppsala, Sweden).

### 4.7. In Vitro Cytotoxicity Analysis

The in vitro toxicity assay was performed using SKOV3 and OVCAR3 cells. The cells were seeded in 96-well plates at a density of 5000 cells per well and incubated overnight to allow attachment. A decreasing concentration series of Ec1-LoPE was prepared with serial dilution in culture medium and added to the cells after removal of cell medium (*n* = 4−6). The 96-well plates were incubated at 37 °C, 5% CO2 in a humidified incubator for 72 h. Cell viability was measured using cell counting kit-8 (CCK-8; Sigma-Aldrich) according to the manufacturer’s protocol. In brief, after 72 h the incubation medium was replaced with fresh medium and 10 μL of CCK-8 solution was added into each well followed by additional incubation for 2–4 h. The absorbance (OD value) was measured with a microplate reader at 450 nm. The viability of cells incubated with medium only (without the addition of Ec1-PE) was used as 100% viability control. The relative viability was analyzed by GraphPad Prism (version 8.0; GraphPad software, Inc., La Jolla, CA, USA) using a log(inhibitor) vs. response-variable slope (four parameters) model providing half-maximal inhibitory concentration (IC_50_) values.

### 4.8. Animal Studies

The animal studies were planned and executed following Swedish national legislation on protection of laboratory animals. The experiments with mice were approved by the local ethical committee for animal research in Uppsala, Sweden (permit 5.8.18-11931/2020).

### 4.9. Biodistribution Study of Ec1-LoPE

For implantation of tumors, 10^7^ EpCAM-positive SKOV3 cells or 10^7^ EpCAM-negative Ramos cells in 100 µL of medium were subcutaneously (s.c.) injected on the abdomen (SKOV3) or on the hind leg (Ramos) of female BALB/c nu/nu mice. The biodistribution experiments were performed 24 days after cell inoculation. The average animal weight was 18 ± 1 g and the average tumor size was 0.3 ± 0.2 g.

To evaluate the biodistribution over time, mice bearing SKOV3 xenografts were intravenously (i.v.) injected with 5 μg (10 kBq) of [^125^I]I-PIB-Ec1-LoPE and the biodistribution was measured 4, 24, and 48 h post-injection (p.i.). To study the in vivo specificity, biodistribution was measured in mice bearing Ramos xenografts at 4 h p.i. Three to five animals per data point were used. Before dissection, the mice were weighed and anesthetized by an intraperitoneal (i.p.) injection of a ketamine-xylazine solution (30 μL of solution per gram body weight; ketamine 10 mg/mL; xylazine 1 mg/mL). The animals were sacrificed by heart puncture and blood was collected. Organs and tissues were excised, and their activity and weight were measured. The activity uptake was calculated as the percentage of injected dose per gram of sample (%ID/g).

### 4.10. Experimental Therapy with Ec1-LoPE and Trastuzumab in a SKOV3 Xenograft Model

To evaluate the therapeutic efficacy of EpCAM-targeting Ec1-LoPE in combination with HER2-targeting trastuzumab, forty female BALB/c nu/nu mice were subcutaneously implanted (abdomen) with 10^7^ SKOV3 cells in 100 μL media. One week after the implantation, the mice were randomly distributed to four groups, A–D (10 mice per group). In the control group A, mice were injected s.c. (scruff of the neck) with 100 μL of vehicle (0.5% BSA in PBS) for six consecutive weeks. In trastuzumab treatment group B, mice were s.c. injected with trastuzumab with a loading dose of 8 mg/kg during the first two weeks and then with 4 mg/kg during four consecutive weeks. In toxin treatment group C, mice were i.v. injected with Ec1-LoPE with a dose of 1 mg/kg at days 0, 2, 4, 7, 35, 37, 39, 42. In combination treatment group D, mice were i.v. injected with Ec1-LoPE with the dose of 1 mg/kg at days 0, 2, 4, 7, 35, 37, 39, 42 and with trastuzumab with a loading dose of 8 mg/kg during the first two weeks and then with 4 mg/kg during four consecutive weeks. The tumor volumes at the start of treatment (day 0) were 91 ± 25, 99 ± 42, 72 ± 26, and 70 ± 13 mm^3^ for mice in groups A, B, C, and D, respectively. Throughout the experiment, tumor volumes and body weights were measured twice per week. Tumor size was measured with calipers to record the largest longitudinal (length) and transverse (width) diameter and the tumor volume (V) was calculated using the formula: V = 1/2 × (length × width^2^). Mice were euthanized when the subcutaneous tumor volume exceeded 1000 mm^3^, bleeding sores on the tumor were observed or 15% overall weight loss or 10% weight loss within one week was measured. In agreement with the ethical permit, the study was terminated 90 days after starting the injections. When the mice were sacrificed, livers and kidneys were collected, formalin fixed and embedded in paraffin. Staining with hematoxylin and eosin was performed using standard procedures. The stained samples were investigated for histopathologic changes. The histologic evaluation was performed at the Department of Pathology and Wildlife Diseases, National Veterinary Institute, Uppsala, Sweden.

### 4.11. Imaging of HER2 and EpCAM Expression

Whole-body SPECT/CT scans of mice bearing SKOV3 xenografts were performed using nanoScan SPECT/CT (Mediso Medical Imaging Systems, Budapest, Hungary). For imaging of EpCAM expression, mice from control group A were injected with [^125^I]I-PIB-Ec1 (10 μg, 2.2 MBq). At 6 h pi, mice were anesthetized, and images were acquired for 20 min. For imaging of HER2 expression, mice from control group A were injected with [^99m^Tc]Tc-labeled ZHER2:V2 affibody molecule (5 μg, 30 MBq) and images were acquired at 4 h pi for 10 min. Detailed imaging and reconstruction protocol is provided elsewhere [90].

To validate imaging results, a multiplex immunohistochemistry test was performed to analyze EpCAM and HER2 expression in SKOV-3 xenografts. Tumor tissue was fixed in 10% formalin solution and embedded to paraffin according standard operating procedure. Multiplex IHC was performed with a Bond RXm system (Leica, Hamburg, Germany) with antibodies against EpCAM (ab71916, rabbit, polyclonal, 1:1000, Abcam, Cambridge, UK; detected by Opal 570) and HER2 (A0485, rabbit, polyclonal, 1:900, Agilent, Santa-Clara, CA, USA; detected by Opal 690). Protein blocking was performed using 3% BSA-PBS (Sigma, St. Louis, MO, USA). TSA visualization was performed with the Opal seven-color IHC kit (Akoya Bio, Marlborough, MA, USA). Nuclei were counterstained with DAPI and slides were enclosed in fluorescence mounting medium (Agilent, Santa-Clara, CA, USA). Slides were scanned using the Vectra 3.0 (PerkinElmer, Waltham, MA, USA). Tissue imaging and analyses was performed using inForm Advanced Image Analysis software (inForm 2.2.1; Perkin Elmer, Waltham, MA, USA).

### 4.12. Statistics

Statistical analysis was performed using GraphPad Prism (version 8.0; GraphPad Software, Inc., La Jolla, CA, USA). A *p*-value < 0.05 was considered a statistically significant difference. Equal variance was assumed for each analysis. The in vitro specificity and cellular processing data are presented as the mean ± standard deviation (SD) of three samples, the cytotoxicity data- of four to six samples. The data were analyzed using an unpaired two-tailed *t*-test. In the therapy experiments, groups of ten mice were used. Comparison of variation of tumor size at each time point between the groups in therapy study was performed by one-way ANOVA with Bonferroni’s multiple comparisons test based on a single measurement. Difference in survival was evaluated using Mantel–Cox log-rank test. Therapy outcomes were analyzed using chi-square test.

## 5. Conclusions

Ec1-LoPE has specific binding to tumors and provides an anti-tumor effect in EpCAM-expressing SKOV3 xenografts. Its combination with trastuzumab provides an additive effect and improves survival of trastuzumab-treated mice bearing EpCAM and HER2-expressing SKOV3 xenografts. The treatment with Ec1-LoPE was tolerated well without signs of hepatic or kidney toxicity after repeated administration of doses up to 8 mg/kg. Ec1-LoPE might be a potential adjuvant therapeutic strategy for HER2-positive metastatic cancers.

## Figures and Tables

**Table 1:** Equilibrium dissociation constants (K_D_) for the interaction between [^125^I]I-Ec1-LoPE and OVCAR3 and SKOV3 cells.

Cell Line	K_D1_ (pM)	%	K_D2_ (nM)	%
SKOV3 (*n* = 2)	833 ± 168	59 ± 2	53 ± 4	25 ± 4
OVCAR3 (*n* = 2)	305 ± 33	79 ± 1	26 ± 1	3 ± 2

## Data Availability

The data generated during the current study are available from the corresponding authors upon reasonable request.

## Supplementary Materials

The following are available online at https://www.mdpi.com/article/10.3390/cancers13163939/s1, Figure S1: One-way ANOVA analysis Bonferroni’s multiple comparisons test of tumor volumes at the treatment start (day 0) and four days after treatment start. *p*-values for each comparison are presented. Each group included 10 mice. Figure S2. Relative tumor growth. Tumor volumes for each animals were normalized to their volume at treatment start (day 0). Each type of treatment was applied for group of 10 mice. Figure S3. Results of histopathological examination. Formalin-fixed paraffin-embedded samples was stained with hematoxylin and eosin. Upper raw: Normal hepatocytes with occasional dividing cells in treated groups (arrow) (×60). Middle raw: Renal cortex with scattered glomeruli and proximal and distal tubules (×20). Bottom raw: Tumors Ec1-LoPE: a small nodule consisting of tumor cells surrounded by an inflammatory cell infiltrate and fibrosis. The vast majority of tumor cells in this nodule is considered to be live cells. Arrows show pleomorphic, viable tumor cells. Ec1-LoPE and trastuzumab: Irregularly shaped tumor cells are arranged in irregular follicles with tendency to solid pattern. Arrows point at cell divisions (×40).

## References

[1] Colombo N., Sessa C., du Bois A., Ledermann J., McCluggage W.G., McNeish I., Morice P., Pignata S., Ray-Coquard I., ESMO-ESGO Ovarian Cancer Consensus Conference Working Group (2019). ESMO-ESGO consensus conference recommendations on ovarian cancer: Pathology and molecular biology, early and advanced stages, borderline tumours and recurrent disease. Ann. Oncol..

[2] Bast R.C., Hennessy B., Mills G.B. (2009). The biology of ovarian cancer: New opportunities for translation. Nat. Rev. Cancer..

[3] Patel A., Iyer P., Matsuzaki S., Matsuo K., Sood A.K., Fleming N.D. (2021). Emerging Trends in Neoadjuvant Chemotherapy for Ovarian Cancer. Cancers.

[4] Köbel M., Kalloger S.E., Boyd N., McKinney S., Mehl E., Palmer C., Leung S., Bowen N.J., Ionescu D.N., Rajput A. (2008). Ovarian carcinoma subtypes are different diseases: Implications for biomarker studies. PLoS Med..

[5] Pils D., Pinter A., Reibenwein J., Alfanz A., Horak P., Schmid B.C., Hefler L., Horvat R., Reinthaller A., Zeillinger R. (2007). In ovarian cancer the prognostic influence of HER2/neu is not dependent on the CXCR4/SDF-1 signalling pathway. Br. J. Cancer.

[6] Spizzo G., Fong D., Wurm M., Ensinger C., Obrist P., Hofer C., Mazzoleni G., Gastl G., Went P. (2011). EpCAM expression in primary tumour tissues and metastases: An immunohistochemical analysis. J. Clin. Pathol..

[7] Hodeib M., Serna-Gallegos T., Tewari K.S. (2015). A review of HER2-targeted therapy in breast and ovarian cancer: Lessons from antiquity—CLEOPATRA and PENELOPE. Future Oncol..

[8] Oh D.Y., Bang Y.J. (2020). HER2-targeted therapies—A role beyond breast cancer. Nat. Rev. Clin. Oncol..

[9] Bang Y.J., Van Cutsem E., Feyereislova A., Chung H.C., Shen L., Sawaki A., Lordick F., Ohtsu A., Omuro Y., Satoh T. (2010). ToGA Trial Investigators. Trastuzumab in combination with chemotherapy versus chemotherapy alone for treatment of HER2-positive advanced gastric or gastro-oesophageal junction cancer (ToGA): A phase 3, open-label, randomised controlled trial. Lancet.

[10] Slamon D., Eiermann W., Robert N., Pienkowski T., Martin M., Press M., Mackey J., Glaspy J., Chan A., Pawlicki M. (2011). Breast Cancer International Research Group. Adjuvant trastuzumab in HER2-positive breast cancer. N. Engl. J. Med..

[11] Giordano S.H., Temin S., Kirshner J.J., Chandarlapaty S., Crews J.R., Davidson N.E., Esteva F.J., Gonzalez-Angulo A.M., Krop I., Levinson J. (2014). American Society of Clinical Oncology. Systemic Therapy for Patients with Advanced Human Epidermal Growth Factor Receptor 2-Positive Breast Cancer: American Society of Clinical Oncology Clinical Practice Guideline. J. Clin. Oncol..

[12] Tai W., Mahato R., Cheng K. (2010). The role of HER2 in cancer therapy and targeted drug delivery. J. Control. Release..

[13] Dokmanovic M., Wu W.J. (2015). Monitoring Trastuzumab Resistance and Cardiotoxicity: A Tale of Personalized Medicine. Adv. Clin. Chem..

[14] Goutsouliak K., Veeraraghavan J., Sethunath V., De Angelis C., Osborne C.K., Rimawi M.F., Schiff R. (2020). Towards personalized treatment for early stage HER2-positive breast cancer. Nat. Rev. Clin. Oncol..

[15] Bon G., Pizzuti L., Laquintana V., Loria R., Porru M., Marchiò C., Krasniqi E., Barba M., Maugeri-Saccà M., Gamucci T. (2020). Loss of HER2 and decreased T-DM1 efficacy in HER2 positive advanced breast cancer treated with dual HER2 blockade: The SePHER Study. J. Exp. Clin. Cancer Res..

[16] Gebhart G., Lamberts L.E., Wimana Z., Garcia C., Emonts P., Ameye L., Stroobants S., Huizing M., Aftimos P., Tol J. (2016). Molecular imaging as a tool to investigate heterogeneity of advanced HER2-positive breast cancer and to predict patient outcome under trastuzumab emtansine (T-DM1): The ZEPHIR trial. Ann. Oncol..

[17] Dollé L., Theise N.D., Schmelzer E., Boulter L., Gires O., van Grunsven L.A. (2015). EpCAM and the biology of hepatic stem/progenitor cells. Am. J. Physiol. Gastrointest. Liver Physiol..

[18] Mohtar M.A., Syafruddin S.E., Nasir S.N., Low T.Y. (2020). Revisiting the Roles of Pro-Metastatic EpCAM in Cancer. Biomolecules.

[19] Fagotto F., Aslemarz A. (2020). EpCAM cellular functions in adhesion and migration, and potential impact on invasion: A critical review. Biochim. Biophys. Acta Rev. Cancer..

[20] Gires O., Pan M., Schinke H., Canis M., Baeuerle P.A. (2020). Expression and function of epithelial cell adhesion molecule EpCAM: Where are we after 40 years?. Cancer Metastasis Rev..

[21] Bellone S., Siegel E.R., Cocco E., Cargnelutti M., Silasi D.A., Azodi M., Schwartz P.E., Rutherford T.J., Pecorelli S., Santin A.D. (2009). Overexpression of epithelial cell adhesion molecule in primary, metastatic, and recurrent/chemotherapy-resistant epithelial ovarian cancer: Implications for epithelial cell adhesion molecule-specific immunotherapy. Int. J. Gynecol. Cancer.

[22] Zheng J., Wang Y., Zhao L., Zhao S., Cui M. (2017). Overexpression of CD44 and EpCAM may be associated with the initiation and progression of epithelial ovarian cancer. Int. J. Clin. Exp. Pathol..

[23] Spizzo G., Went P., Dirnhofer S., Obrist P., Moch H., Baeuerle P.A., Mueller-Holzner E., Marth C., Gastl G., Zeimet A.G. (2006). Overexpression of epithelial cell adhesion molecule (Ep-CAM) is an independent prognostic marker for reduced survival of patients with epithelial ovarian cancer. Gynecol. Oncol..

[24] Wimberger P., Gilet H., Gonschior A.K., Heiss M.M., Moehler M., Oskay-Oezcelik G., Al-Batran S.E., Schmalfeldt B., Schmittel A., Schulze E. (2012). Deterioration in quality of life (QoL) in patients with malignant ascites: Results from a phase II/III study comparing paracentesis plus catumaxomab with paracentesis alone. Ann. Oncol..

[25] ClinicalTrials.gov (2005). Identifier NCT00635596, Phase I Study of MT110 in Lung Cancer (Adenocarcinoma and Small Cell), Gastric Cancer or Adenocarcinoma of the Gastro-Esophageal Junction, Colorectal Cancer, Breast Cancer, Hormone-Refractory Prostate Cancer, and Ovarian Cancer (MT110-101).

[26] Tayama S., Motohara T., Narantuya D., Li C., Fujimoto K., Sakaguchi I., Tashiro H., Saya H., Nagano O., Katabuchi H. (2017). The impact of EpCAM expression on response to chemotherapy and clinical outcomes in patients with epithelial ovarian cancer. Oncotarget.

[27] Wei X., Dombkowski D., Meirelles K., Pieretti-Vanmarcke R., Szotek P.P., Chang H.L., Preffer F.I., Mueller P.R., Teixeira J. (2010). Mullerian inhibiting substance preferentially inhibits stem/progenitors in human ovarian cancer cell lines compared with chemotherapeutics. Proc. Natl. Acad. Sci. USA.

[28] Walters Haygood C.L., Arend R.C., Straughn J.M., Buchsbaum D.J. (2014). Ovarian cancer stem cells: Can targeted therapy lead to improved progression-free survival?. World J. Stem Cells.

[29] Schmidt M., Scheulen M.E., Dittrich C., Obrist P., Marschner N., Dirix L., Schmidt M., Rüttinger D., Schuler M., Reinhardt C. (2010). An open-label, randomized phase II study of adecatumumab, a fully human anti-EpCAM antibody, as monotherapy in patients with metastatic breast cancer. Ann. Oncol..

[30] Heiss M.M., Murawa P., Koralewski P., Kutarska E., Kolesnik O.O., Ivanchenko V.V., Dudnichenko A.S., Aleknaviciene B., Razbadauskas A., Gore M. (2010). The trifunctional antibody catumaxomab for the treatment of malignant ascites due to epithelial cancer: Results of a prospective randomized phase II/III trial. Int. J. Cancer.

[31] Eyvazi S., Farajnia S., Dastmalchi S., Kanipour F., Zarredar H., Bandehpour M. (2018). Antibody Based EpCAM Targeted Therapy of Cancer, Review and Update. Curr. Cancer Drug Targets.

[32] Seimetz D., Lindhofer H., Bokemeyer C. (2010). Development and approval of the trifunctional antibody catumaxomab (anti-EpCAM x anti-CD3) as a targeted cancer immunotherapy. Cancer Treat. Rev..

[33] Knödler M., Körfer J., Kunzmann V., Trojan J., Daum S., Schenk M., Kullmann F., Schroll S., Behringer D., Stahl M. (2018). Randomised phase II trial to investigate catumaxomab (anti-EpCAM × anti-CD3) for treatment of peritoneal carcinomatosis in patients with gastric cancer. Br. J. Cancer..

[34] Schmidt M., Rüttinger D., Sebastian M., Hanusch C.A., Marschner N., Baeuerle P.A., Wolf A., Göppel G., Oruzio D., Schlimok G. (2012). Phase IB study of the EpCAM antibody adecatumumab combined with docetaxel in patients with EpCAM-positive relapsed or refractory advanced-stage breast cancer. Ann. Oncol..

[35] Macdonald J., Henri J., Roy K., Hays E., Bauer M., Veedu R.N., Pouliot N., Shigdar S. (2018). EpCAM Immunotherapy versus Specific Targeted Delivery of Drugs. Cancers.

[36] Andersson Y., Engebraaten O., Juell S., Aamdal S., Brunsvig P., Fodstad Ø., Dueland S. (2015). Phase I trial of EpCAM-targeting immunotoxin MOC31PE, alone and in combination with cyclosporin. Br. J. Cancer.

[37] Frøysnes I.S., Andersson Y., Larsen S.G., Davidson B., Øien J.T., Olsen K.H., Giercksky K.E., Julsrud L., Fodstad Ø., Dueland S. (2017). Novel Treatment with Intraperitoneal MOC31PE Immunotoxin in Colorectal Peritoneal Metastasis: Results From the ImmunoPeCa Phase 1 Trial. Ann. Surg. Oncol..

[38] Cizeau J., Grenkow D.M., Brown J.G., Entwistle J., MacDonald G.C. (2009). Engineering and biological characterization of VB6-845, an anti-EpCAM immunotoxin containing a T-cell epitope-depleted variant of the plant toxin bouganin. J. Immunother..

[39] Di Paolo C., Willuda J., Kubetzko S., Lauffer I., Tschudi D., Waibel R., Plückthun A., Stahel R.A., Zangemeister-Wittke U. (2003). A recombinant immunotoxin derived from a humanized epithelial cell adhesion molecule-specific single-chain antibody fragment has potent and selective antitumor activity. Clin. Cancer Res..

[40] MacDonald G.C., Rasamoelisolo M., Entwistle J., Cuthbert W., Kowalski M., Spearman M.A., Glover N. (2009). A phase I clinical study of intratumorally administered VB4-845, an anti-epithelial cell adhesion molecule recombinant fusion protein, in patients with squamous cell carcinoma of the head and neck. Med. Oncol..

[41] Plückthun A. (2015). Designed ankyrin repeat proteins (DARPins): Binding proteins for research, diagnostics, and therapy. Annu. Rev. Pharmacol. Toxicol..

[42] Sokolova E., Proshkina G., Kutova O., Shilova O., Ryabova A., Schulga A., Stremovskiy O., Zdobnova T., Balalaeva I., Deyev S. (2016). Recombinant targeted toxin based on HER2-specific DARPin possesses a strong selective cytotoxic effect in vitro and a potent antitumor activity in vivo. J. Control. Release.

[43] Sokolova E.A., Shilova O.N., Kiseleva D.V., Schulga A.A., Balalaeva I.V., Deyev S.M. (2019). HER2-Specific Targeted Toxin DARPin-LoPE: Immunogenicity and Antitumor Effect on Intraperitoneal Ovarian Cancer Xenograft Model. Int. J. Mol. Sci..

[44] Souied E.H., Devin F., Mauget-Faÿsse M., Kolář P., Wolf-Schnurrbusch U., Framme C., Gaucher D., Querques G., Stumpp M.T., Wolf S. (2014). Treatment of exudative age-related macular degeneration with a designed ankyrin repeat protein that binds vascular endothelial growth factor: A phase I/II study. Am. J. Ophthalmol..

[45] Tolmachev V., Bragina O., Schulga A., Konovalova E., Garbukov E., Vorobyeva A., Orlova A., Deyev S., Chernov V. (2021). First-in-human Evaluation of [99mTc]Tc-(HE)3-G3, a Novel DARPin-Based Agent for Imaging of HER2 Expression in Breast Cancer. Nucl. Med. Biol..

[46] Goldstein R., Sosabowski J., Livanos M., Leyton J., Vigor K., Bhavsar G., Nagy-Davidescu G., Rashid M., Miranda E., Yeung J. (2015). Development of the designed ankyrin repeat protein (DARPin) G3 for HER2 molecular imaging. Eur. J. Nucl. Med. Mol. Imaging.

[47] Deyev S., Vorobyeva A., Schulga A., Proshkina G., Güler R., Löfblom J., Mitran B., Garousi J., Altai M., Buijs J. (2019). Comparative Evaluation of Two DARPin Variants: Effect of Affinity, Size, and Label on Tumor Targeting Properties. Mol. Pharm..

[48] Vorobyeva A., Sсhulga A., Konovalova E., Güler R., Mitran B., Garousi J., Rinne S., Löfblom J., Orlova A., Deyev S. (2019). Comparison of tumor-targeting properties of directly and indirectly radioiodinated designed ankyrin repeat protein (DARPin) G3 variants for molecular imaging of HER2. Int. J. Oncol..

[49] Vorobyeva A., Schulga A., Rinne S.S., Günther T., Orlova A., Deyev S., Tolmachev V. (2019). Indirect Radioiodination of DARPin G3 Using N-succinimidyl-Para-Iodobenzoate Improves the Contrast of HER2 Molecular Imaging. Int. J. Mol. Sci..

[50] Deyev S.M., Vorobyeva A., Schulga A., Abouzayed A., Günther T., Garousi J., Konovalova E., Ding H., Gräslund T., Orlova A. (2020). Effect of a radiolabel biochemical nature on tumor-targeting properties of EpCAM-binding engineered scaffold protein DARPin Ec1. Int. J. Biol. Macromol..

[51] Vorobyeva A., Konovalova E., Xu T., Schulga A., Altai M., Garousi J., Rinne S.S., Orlova A., Tolmachev V., Deyev S. (2020). Feasibility of Imaging EpCAM Expression in Ovarian Cancer Using Radiolabeled DARPin Ec1. Int. J. Mol. Sci..

[52] Vorobyeva A., Bezverkhniaia E., Konovalova E., Schulga A., Garousi J., Vorontsova O., Abouzayed A., Orlova A., Deyev S., Tolmachev V. (2020). Radionuclide Molecular Imaging of EpCAM Expression in Triple-Negative Breast Cancer Using the Scaffold Protein DARPin Ec1. Molecules.

[53] Stefan N., Martin-Killias P., Wyss-Stoeckle S., Honegger A., Zangemeister-Wittke U., Plückthun A. (2011). DARPins recognizing the tumor-associated antigen EpCAM selected by phage and ribosome display and engineered for multivalency. J. Mol. Biol..

[54] Shramova E., Proshkina G., Shipunova V., Ryabova A., Kamyshinsky R., Konevega A., Schulga A., Konovalova E., Telegin G., Deyev S. (2020). Dual Targeting of Cancer Cells with DARPin-Based Toxins for Overcoming Tumor Escape. Cancers.

[55] Hassan R., Bullock S., Premkumar A., Kreitman R.J., Kindler H., Willingham M.C., Pastan I. (2007). Phase I study of SS1P, a recombinant anti-mesothelin immunotoxin given as a bolus I.V. infusion to patients with mesothelin-expressing mesothelioma, ovarian, and pancreatic cancers. Clin. Cancer Res..

[56] Kreitman R.J., Stetler-Stevenson M., Margulies I., Noel P., Fitzgerald D.J., Wilson W.H., Pastan I. (2009). Phase II trial of recombinant immunotoxin RFB4(dsFv)-PE38 (BL22) in patients with hairy cell leukemia. J. Clin. Oncol..

[57] Kowalski M., Entwistle J., Cizeau J., Niforos D., Loewen S., Chapman W., MacDonald G.C. (2010). A Phase I study of an intravesically administered immunotoxin targeting EpCAM for the treatment of nonmuscle-invasive bladder cancer in BCGrefractory and BCG-intolerant patients. Drug Des. Devel. Ther..

[58] Weldon J.E., Pastan I. (2011). A guide to taming a toxin--recombinant immunotoxins constructed from Pseudomonas exotoxin A for the treatment of cancer. FEBS J..

[59] Liu W., Onda M., Lee B., Kreitman R.J., Hassan R., Xiang L., Pastan I. (2012). Recombinant immunotoxin engineered for low immunogenicity and antigenicity by identifying and silencing human B-cell epitopes. Proc. Natl. Acad. Sci. USA.

[60] Proshkina G.M., Kiseleva D.V., Shilova O.N., Ryabova A.V., Shramova E.I., Stremovskiy O.A., Deyev S.M. (2017). Bifunctional Toxin DARP-LoPE Based on the Her2-Specific Innovative Module of a Non-Immunoglobulin Scaffold as a Promising Agent for Theranostics. Mol. Biol..

[61] Lopez J.S., Banerji U. (2017). Combine and conquer: Challenges for targeted therapy combinations in early phase trials. Nat. Rev. Clin. Oncol..

[62] Akbari B., Farajnia S., Ahdi Khosroshahi S., Safari F., Yousefi M., Dariushnejad H., Rahbarnia L. (2017). Immunotoxins in cancer therapy: Review and update. Int. Rev. Immunol..

[63] Giordano S.H., Temin S., Chandarlapaty S., Crews J.R., Esteva F.J., Kirshner J.J., Krop I.E., Levinson J., Lin N.U., Modi S. (2018). Systemic Therapy for Patients With Advanced Human Epidermal Growth Factor Receptor 2-Positive Breast Cancer: ASCO Clinical Practice Guideline Update. J. Clin. Oncol..

[64] Brinkmann U., Kontermann R.E. (2017). The making of bispecific antibodies. MAbs.

[65] Spizzo G., Obrist P., Ensinger C., Theurl I., Dünser M., Ramoni A., Gunsilius E., Eibl G., Mikuz G., Gastl G. (2002). Prognostic significance of Ep-CAM AND Her-2/neu overexpression in invasive breast cancer. Int. J. Cancer.

[66] Kloudová K., Hromádková H., Partlová S., Brtnický T., Rob L., Bartůňková J., Hensler M., Halaška M.J., Špíšek R., Fialová A. (2016). Expression of tumor antigens on primary ovarian cancer cells compared to established ovarian cancer cell lines. Oncotarget.

[67] Anglesio M.S., Kommoss S., Tolcher M.C., Clarke B., Galletta L., Porter H., Damaraju S., Fereday S., Winterhoff B.J., Kalloger S.E. (2013). Molecular characterization of mucinous ovarian tumours supports a stratified treatment approach with HER2 targeting in 19% of carcinomas. J. Pathol..

[68] Gorringe K.L., Cheasley D., Wakefield M.J., Ryland G.L., Allan P.E., Alsop K., Amarasinghe K.C., Ananda S., Bowtell D.D.L., Christie M. (2020). Therapeutic options for mucinous ovarian carcinoma. Gynecol. Oncol..

[69] Tan D.S., Iravani M., McCluggage W.G., Lambros M.B., Milanezi F., Mackay A., Gourley C., Geyer F.C., Vatcheva R., Millar J. (2011). Genomic analysis reveals the molecular heterogeneity of ovarian clear cell carcinomas. Clin. Cancer. Res..

[70] Stish B.J., Chen H., Shu Y., Panoskaltsis-Mortari A., Vallera D.A. (2007). Increasing anticarcinoma activity of an anti-erbB2 recombinant immunotoxin by the addition of an anti-EpCAM sFv. Clin. Cancer Res..

[71] Frankel A.E., Kreitman R.J., Sausville E.A. (2000). Targeted toxins. Clin. Cancer Res..

[72] Kondo T., FitzGerald D., Chaudhary V.K., Adhya S., Pastan I. (1988). Activity of immunotoxins constructed with modified Pseudomonas exotoxin A lacking the cell recognition domain. J. Biol. Chem..

[73] Siegall C.B., Chaudhary V.K., FitzGerald D.J., Pastan I. (1989). Functional analysis of domains II, Ib, and III of Pseudomonas exotoxin. J. Biol. Chem..

[74] Kreitman R.J., Squires D.R., Stetler-Stevenson M., Noel P., FitzGerald D.J., Wilson W.H., Pastan I. (2005). Phase I trial of recombinant immunotoxin RFB4(dsFv)-PE38 (BL22) in patients with B-cell malignancies. J. Clin. Oncol..

[75] Posey J.A., Khazaeli M.B., Bookman M.A., Nowrouzi A., Grizzle W.E., Thornton J., Carey D.E., Lorenz J.M., Sing A.P., Siegall C.B. (2002). A phase I trial of the single-chain immunotoxin SGN-10 (BR96 sFv-PE40) in patients with advanced solid tumors. Clin. Cancer Res..

[76] MacDonald G.C., Rasamoelisolo M., Entwistle J., Cizeau J., Bosc D., Cuthbert W., Kowalski M., Spearman M., Glover N. (2009). A phase I clinical study of VB4-845: Weekly intratumoral administration of an anti-EpCAM recombinant fusion protein in patients with squamous cell carcinoma of the head and neck. Drug Des. Devel. Ther..

[77] Martin-Killias P., Stefan N., Rothschild S., Plückthun A., Zangemeister-Wittke U. (2011). A novel fusion toxin derived from an EpCAM-specific designed ankyrin repeat protein has potent antitumor activity. Clin. Cancer Res..

[78] Simon M., Stefan N., Borsig L., Plückthun A., Zangemeister-Wittke U. (2014). Increasing the antitumor effect of an EpCAM-targeting fusion toxin by facile click PEGylation. Mol. Cancer Ther..

[79] Stefan N., Zimmermann M., Simon M., Zangemeister-Wittke U., Plückthun A. (2014). Novel prodrug-like fusion toxin with protease-sensitive bioorthogonal PEGylation for tumor targeting. Bioconjug. Chem..

[80] FitzGerald D.J., Wayne A.S., Kreitman R.J., Pastan I. (2011). Treatment of hematologic malignancies with immunotoxins and antibody-drug conjugates. Cancer Res..

[81] Mazor R., Vassall A.N., Eberle J.A., Beers R., Weldon J.E., Venzon D.J., Tsang K.Y., Benhar I., Pastan I. (2012). Identification and elimination of an immunodominant T-cell epitope in recombinant immunotoxins based on Pseudomonas exotoxin A. Proc. Natl. Acad. Sci. USA.

[82] Liu H., Seijsing J., Frejd F.Y., Tolmachev V., Gräslund T. (2015). Target-specific cytotoxic effects on HER2-expressing cells by the tripartite fusion toxin ZHER2:2891-ABD-PE38X8, including a targeting affibody molecule and a half-life extension domain. Int. J. Oncol..

[83] Altai M., Liu H., Orlova A., Tolmachev V., Gräslund T. (2016). Influence of molecular design on biodistribution and targeting properties of an Affibody-fused HER2-recognising anticancer toxin. Int. J. Oncol..

[84] Van der Gun B.T., de Groote M.L., Kazemier H.G., Arendzen A.J., Terpstra P., Ruiters M.H., McLaughlin P.M., Rots M.G. (2011). Transcription factors and molecular epigenetic marks underlying EpCAM overexpression in ovarian cancer. Br. J. Cancer.

[85] Altai M., Liu H., Ding H., Mitran B., Edqvist P.H., Tolmachev V., Orlova A., Gräslund T. (2018). Affibody-derived drug conjugates: Potent cytotoxic molecules for treatment of HER2 over-expressing tumors. J. Control. Release.

[86] Xu T., Ding H., Vorobyeva A., Oroujeni M., Orlova A., Tolmachev V., Gräslund T. (2020). Drug Conjugates Based on a Monovalent Affibody Targeting Vector Can Efficiently Eradicate HER2 Positive Human Tumors in an Experimental Mouse Model. Cancers.

[87] Vorobyeva A., Bragina O., Altai M., Mitran B., Orlova A., Shulga A., Proshkina G., Chernov V., Tolmachev V., Deyev S. (2018). Comparative Evaluation of Radioiodine and Technetium-Labeled DARPin 9_29 for Radionuclide Molecular Imaging of HER2 Expression in Malignant Tumors. Contrast Media Mol. Imaging.

[88] Wållberg H., Orlova A., Altai M., Hosseinimehr S.J., Widström C., Malmberg J., Ståhl S., Tolmachev V. (2011). Molecular design and optimization of 99mTc-labeled recombinant affibody molecules improves their biodistribution and imaging properties. J. Nucl. Med..

[89] Wållberg H., Orlova A. (2008). Slow internalization of anti-HER2 synthetic affibody monomer 111In-DOTA-ZHER2:342-pep2: Implications for development of labeled tracers. Cancer Biother. Radiopharm..

[90] Deyev S.M., Xu T., Liu Y., Schulga A., Konovalova E., Garousi J., Rinne S.S., Larkina M., Ding H., Graslund T. (2021). Influence of the position and composition of radiometals and radioiodine labels on imaging of EpCAM expression in prostate cancer model using the DARPin Ec1. Cancers.

