# Supplementary material for: Imaging-Guided Therapy Simultaneously Targeting HER2 and EpCAM with Trastuzumab and EpCAM-Directed Toxin Provides Additive Effect in Ovarian Cancer Model
Source: Cancers (Basel). 2021 Aug 4;13(16):3939. doi: 10.3390/cancers13163939 (PMC8393281; doi:10.3390/cancers13163939)
Supplement: Supplementary file 1 [file cancers-13-03939-s001.zip › cancers-1269815-supplementary.pdf]

Image-Guided Therapy Simultaneously Targeting HER2 and EpCAM with Trastuzumab and EpCAM-Directed Toxin Provides Additive Effect in Ovarian Cancer Model

Tianqi Xu, Anzhelika Vorobyeva, Alexey Schulga, Elena Konovalova, Olga Vorontsova, Haozhong Ding, Torbjörn Gräslund, Liubov A. Tashireva, Anna Orlova, Vladimir Tolmachev and Sergey M. Deyev

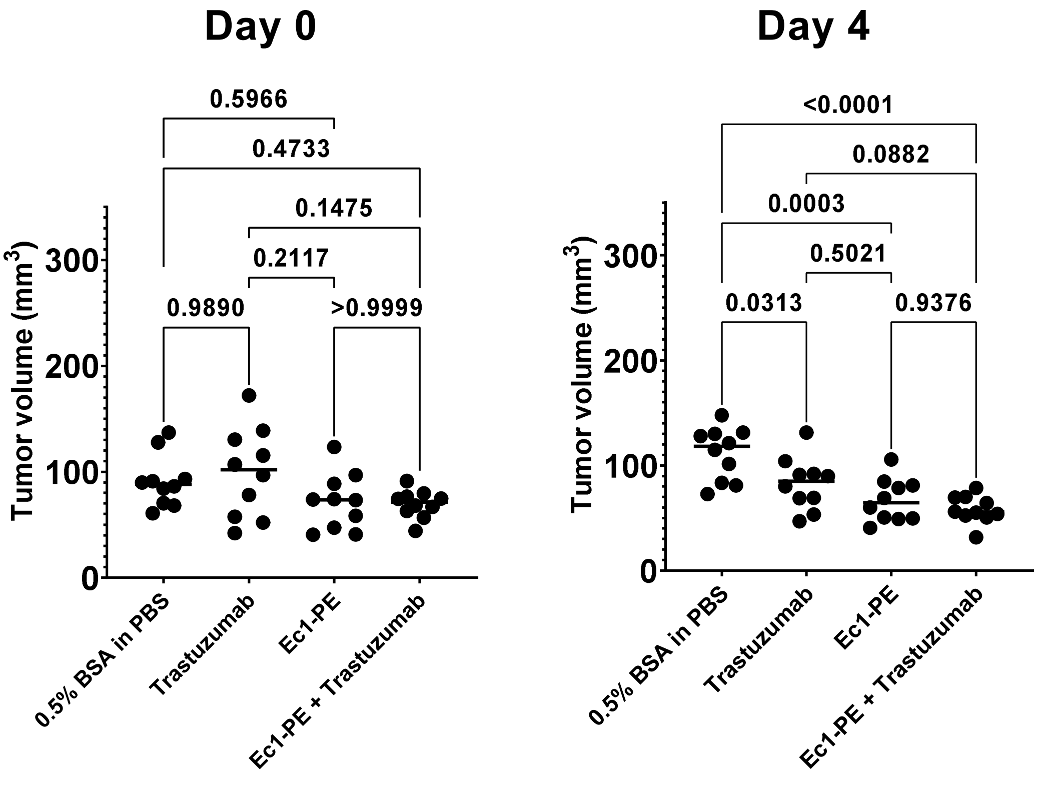

Figure S1. One-way ANOVA analysis Bonferroni's multiple comparisons test of tumor volumes at the treatment start (day 0) and four days after treatment start. P-values for each comparion are presented. Each group included 10 mice.

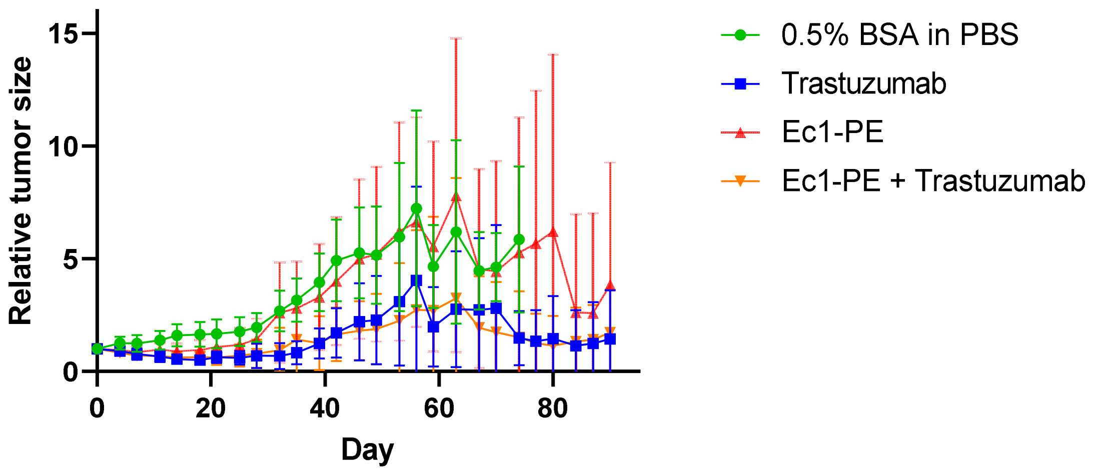

Figure S2. Relative tumor growth. Tumor volumes for each animals were normalized to their volume at treatment start (day 0). Each type of treatment was applied for group of 10 mice.

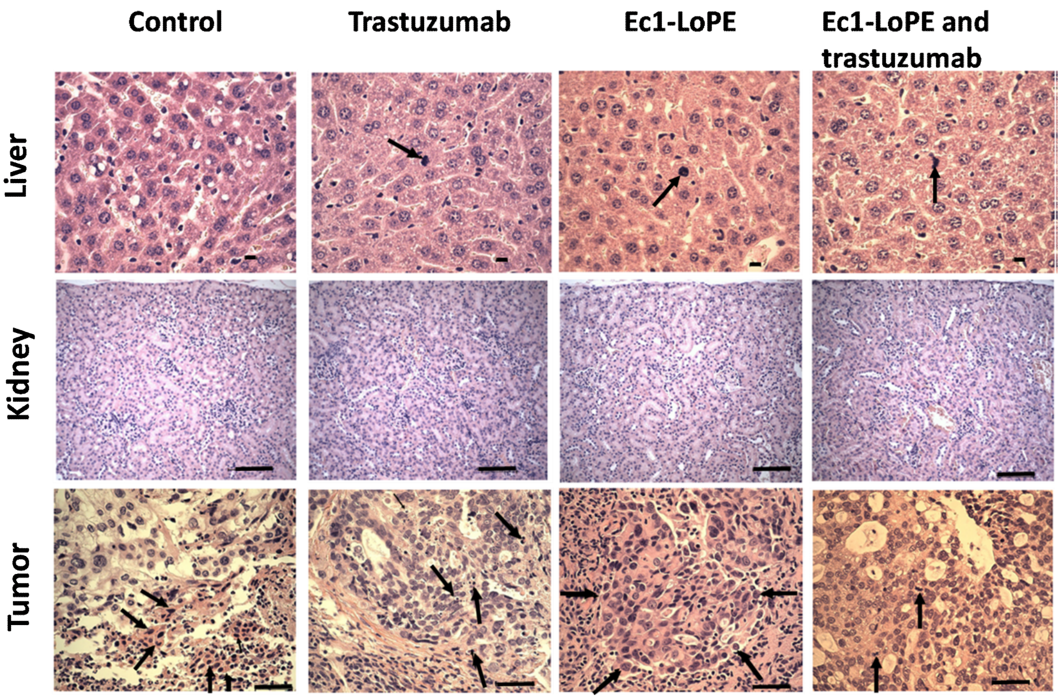

Figure S3. Results of histopathological examination. Formalin-fixed paraffin-embedded samples was stained with hematoxylin and eosin. Upper raw: Normal hepatocytes with occasional dividing cells in treated groups (arrow). ×60. Middle raw: Renal cortex with scattered glomeruli and proximal and distal tubules. × 20. Bottom raw: Tumors Ec1-LoPE: a small nodule consisting of tumor cells surrounded by an inflammatory cell infiltrate and fibrosis. The vast majority of tumor cells in this nodule is considered to be live cells. Arrows show pleomorphic, viable tumor cells. Ec1-LoPE and trastuzumab: Irregularly shaped tumor cells are arranged in irregular follicles with tendency to solid pattern. Arrows point at cell divisions. ×40.
